# A Platform to Build Mobile Health Apps: The Personal Health Intervention Toolkit (PHIT)

**DOI:** 10.2196/mhealth.4202

**Published:** 2015-06-01

**Authors:** Randall Peter Eckhoff, Paul Nicholas Kizakevich, Vesselina Bakalov, Yuying Zhang, Stephanie Patrice Bryant, Maria Ann Hobbs

**Affiliations:** ^1^RTI InternationalResearch Triangle Park, NCUnited States

**Keywords:** intervention studies, mHealth, mobile apps, platform, software engineering, telemedicine, tool, toolkit

## Abstract

Personal Health Intervention Toolkit (PHIT) is an advanced cross-platform software framework targeted at personal self-help research on mobile devices. Following the subjective and objective measurement, assessment, and plan methodology for health assessment and intervention recommendations, the PHIT platform lets researchers quickly build mobile health research Android and iOS apps. They can (1) create complex data-collection instruments using a simple extensible markup language (XML) schema; (2) use Bluetooth wireless sensors; (3) create targeted self-help interventions based on collected data via XML-coded logic; (4) facilitate cross-study reuse from the library of existing instruments and interventions such as stress, anxiety, sleep quality, and substance abuse; and (5) monitor longitudinal intervention studies via daily upload to a Web-based dashboard portal. For physiological data, Bluetooth sensors collect real-time data with on-device processing. For example, using the BinarHeartSensor, the PHIT platform processes the heart rate data into heart rate variability measures, and plots these data as time-series waveforms. Subjective data instruments are user data-entry screens, comprising a series of forms with validation and processing logic. The PHIT instrument library consists of over 70 reusable instruments for various domains including cognitive, environmental, psychiatric, psychosocial, and substance abuse. Many are standardized instruments, such as the Alcohol Use Disorder Identification Test, Patient Health Questionnaire-8, and Post-Traumatic Stress Disorder Checklist. Autonomous instruments such as battery and global positioning system location support continuous background data collection. All data are acquired using a schedule appropriate to the app’s deployment. The PHIT intelligent virtual advisor (iVA) is an expert system logic layer, which analyzes the data in real time on the device. This data analysis results in a tailored app of interventions and other data-collection instruments. For example, if a user anxiety score exceeds a threshold, the iVA might add a meditation intervention to the task list in order to teach the user how to relax, and schedule a reassessment using the anxiety instrument 2 weeks later to re-evaluate. If the anxiety score exceeds a higher threshold, then an advisory to seek professional help would be displayed. Using the easy-to-use PHIT scripting language, the researcher can program new instruments, the iVA, and interventions to their domain-specific needs. The iVA, instruments, and interventions are defined via XML files, which facilities rapid app development and deployment. The PHIT Web-based dashboard portal provides the researcher access to all the uploaded data. After a secure login, the data can be filtered by criteria such as study, protocol, domain, and user. Data can also be exported into a comma-delimited file for further processing. The PHIT framework has proven to be an extensible, reconfigurable technology that facilitates mobile data collection and health intervention research. Additional plans include instrument development in other domains, additional health sensors, and a text messaging notification system.

##  Introduction

With 968 million units sold worldwide, mobile phones accounted for 53.6% of the overall mobile phone sales in 2013 [1]. In the United States in 2013, 56% of adults own mobile phones [2] and 34% own tablets [3]. Because mobile devices have become more prevalent, mobile health care (mHealth) apps will play a growing role for those managing their health concerns [4]. Nielsen’s Connected Life Report from November 2013 indicates that approximately 46 million users in the United States have accessed apps in the fitness and health category, an 18% increase over the previous year [5]. Unfortunately, many of the available mHealth apps are not evidence based. For example, a review of 98 smoking-cessation apps found most to have a low level of adherence to proven methods defined by the US Public Health Service’s Clinical Practice Guidelines for Treating Tobacco Use and Dependence [6].Therefore, mHealth app development and evaluations should be conducted in collaboration with a health researcher who understands the science and can objectively transfer this knowledge to the app developer. Although researchers desire to build quality evidence-based apps to test the mHealth interventions, app development may impose costs of US $50,000-150,000 [7]. For grant-funded research, this can be a significant fraction of project funds, leaving fewer resources for validation studies and efficacy trials. Personal Health Intervention Toolkit (PHIT) helps address this problem by providing a common platform and reusable content for both development and evaluation.

The PHIT platform was conceived to support the PHIT for Duty research projects addressing secondary prevention of psychological and behavioral health problems in persons experiencing symptoms of post-traumatic stress that had not yet risen to the level of a psychological disorder, such as post-traumatic stress disorder (PTSD). In doing this work, we soon realized that a common process model could be derived for mHealth intervention research and implemented in a way to support cost-effective reuse in other health domains and research apps [8]. We therefore set out to design and develop our PHIT framework, with the following goals:

- Creating a common platform from which other mHealth intervention apps can be developed;
- Standardizing how data collection instruments and interventions are implemented, fostering reuse from a common cross-study library; and
- Masking the complexities of software development to reduce development time and enable researchers to focus on the research aims.

This paper describes the PHIT platform and illustrates our high-level programming tool set, which facilitates implementation of mHealth apps through reuse of existing software content and easy development of new content according to study requirements.

## Methods

### PHIT Model

#### Overview

**Figure 1:**
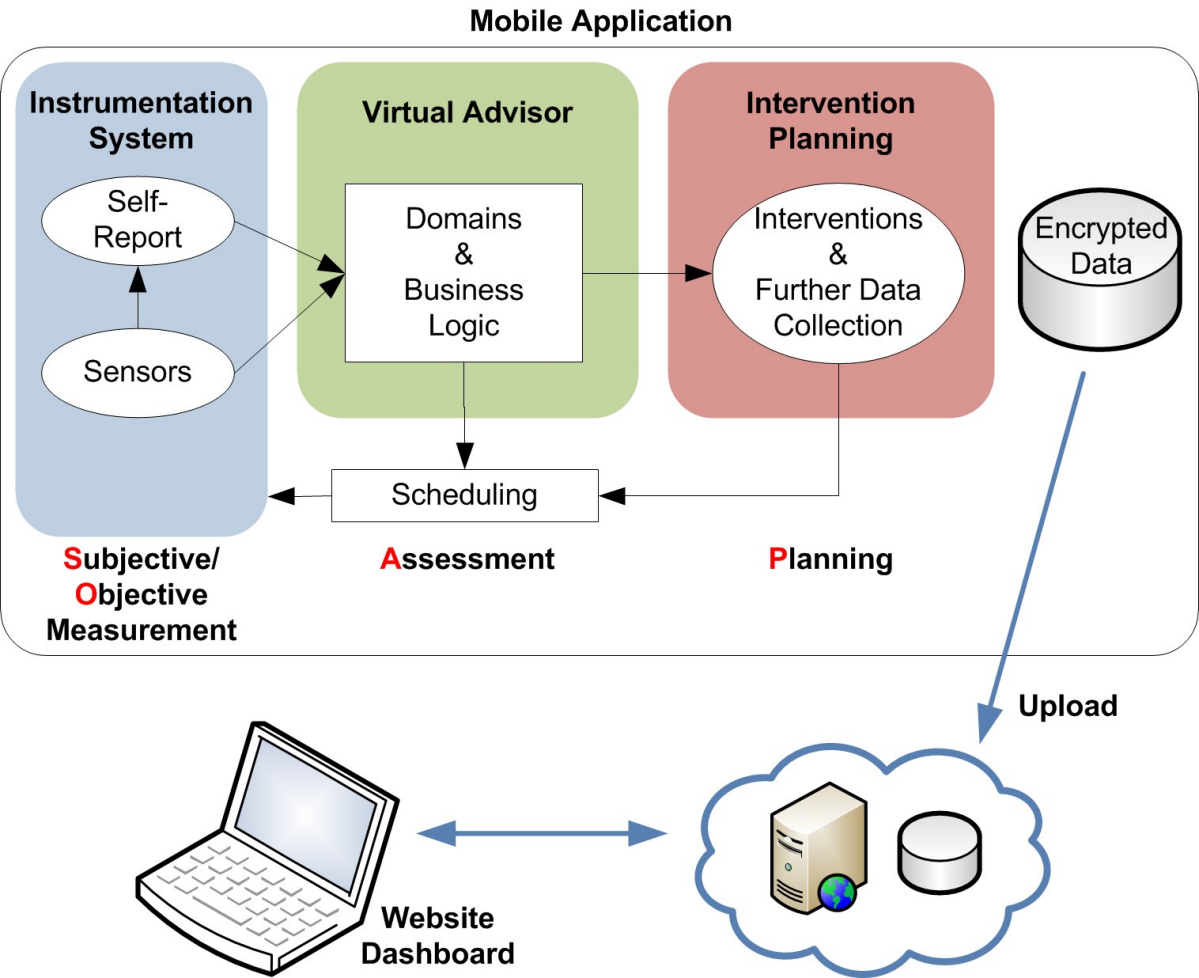
Personal Health Intervention Toolkit model utilizing the subjective, objective, assessment, and plan note methodology.

The PHIT architectural model is based on the subjective and objective measurement, assessment, and plan note methodology [9] for health status analysis, intervention recommendation, self-help activities, and data presentation, creating a feedback loop of personalized health (Figure 1). All data collection, analysis, and planning are performed locally on the mobile device, rather than via Internet services, with secure local data storage. PHIT has the following primary features:

- Integrates self-reported and physiological sensor instruments;
- Analyzes the data in real time on the device via an intelligent virtual advisor (iVA);
- Presents a suite of custom self-help activities and interventions;
- Collects data and adjusts the activities and interventions over time, tailoring the app to the individual needs of each user; and
- Transparently transfers data to a centralized database for conducting data analysis.

Research studies are supported using data objects that tag data with information on the study, protocol, participant identification, and other related information to facilitate analysis. Data access is facilitated using a website dashboard allowing the researcher to monitor the state of longitudinal studies and download study data into comma-separated files for easy analysis. Furthermore, 90% of the app configuration is done via extensible markup language (XML) making it easy to change the behavior of the app.

#### Instrumentation System: Data Collection

The PHIT architecture allows the researcher to reuse data-collection instruments or create their own instruments. Using XML, each instrument has a series of forms (screens) composed of data-collection entities, which represent a data point the researcher wants to collect. For example, a user history instrument may ask for age, weight, and gender information on the first screen and vital signs such as blood pressure and body temperature on the second. These two screens are considered forms in PHIT, and age, weight, gender, systolic and diastolic measures, and temperature are entities. Entities represent the data the researcher is collecting and have two facets to them, namely, (1) the internal, logic side and (2) the graphical user interface. If the app is configured for data storage, the user’s responses are automatically saved. If desired, a researcher may configure the PHIT app to periodically upload data from the local secure database to a backend server, thereby allowing the researcher to examine the collected data and monitor study progress.

The majority of instruments are data-entry instruments, using familiar entities such as text fields, checkboxes, and selection lists. Many of these implement well-established *subjective* health screeners and assessment instruments previously administered through paper forms. Others had been developed ad hoc as needed for a particular study, such as the military deployment history instrument for the PHIT for Duty project. These also can be reused, or adapted as needed, to support new studies. PHIT has a library of over 50 such standardized instruments, including the examples in Table 1.

**Table 1:** Some of the standardized instruments the Personal Health Intervention Toolkit platform has implemented.

Category	Self-report data instrument
Alcohol use	Alcohol Use Disorder Identification Test [10]
Anger	Clinical Anger Scale [11]
Anxiety	General Anxiety Disorder-7 [12]
Combat exposure	Combat Exposure Scale [13]
Coping	Brief Coping Scale [14]
Concussion	Rivermead Post-Concussion Questionnaire [15]
Depression	Patient Health Questionnaire-8 [16-18]
Emotion regulation	Difficulties in Emotion Regulation Scale [19]
Mindfulness	Five Facet Mindfulness Questionnaire [20,21]
Pain	Brief Pain Inventory [22]
Post-traumatic stress disorder	Post-Traumatic Stress Disorder Checklist-Military Version [23]
Resilience	Connor-Davidson Resilience Scale [24]
Sleep quality	Pittsburgh Sleep Quality Index [25,26]
Stress	Perceived Stress Scale-10 [27,28]
Stressors	Impact of Event Scale [29,30]

In addition to the standard data entry fields, radio buttons, and checkboxes, other entities provided by PHIT include audio playback, charts, date picker, image display, Likert scale, and specialty entities, such as game-like cognitive tests (eg, reaction time). These can be combined in various ways for building both data-collection and intervention instruments. For example, following several alcohol reduction-related screens, a series of slides can be combined with audio narration for constructing an integrated multimedia alcohol education instrument.

#### Instrument Coding

**Figure 2:**
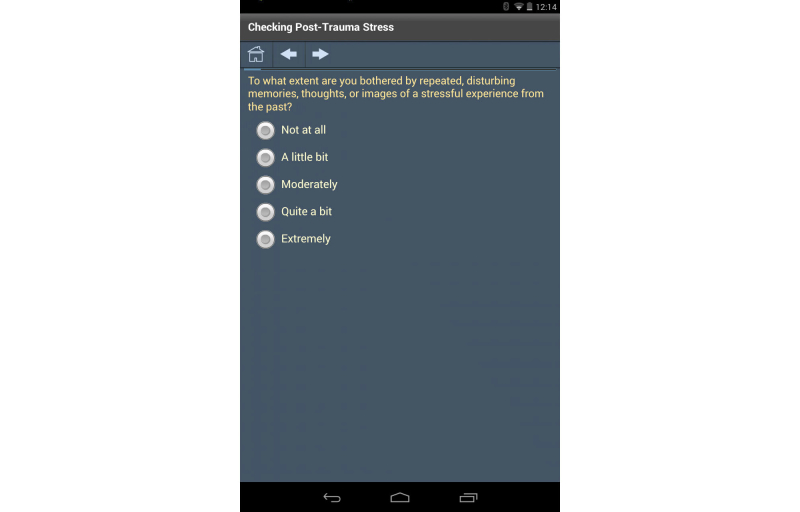
Definition of the first form of the Post-Traumatic Stress Disorder Checklist (PCL) instrument from the Flight Attendant Wellness app.

By coding the entire instrument in XML, the question definitions, codebook responses, data validation, skip logic, and overall flow of the instrument are defined in one place, making it easy to adjust as necessary. The XML definition in Textbox 1 drives the first form from the PTSD checklist instrument used in our “Flight Attendant Wellness” app (Figure 2).

Using simple descriptive text, the form is made up of a single entity, named Q1, comprising a question with a series of radio buttons for the responses. The codebook values are defined in the code attribute of the item element and both the user text and the code are stored in the database. When the user selects one of the five radio buttons, a variable is created as instrumentName_entityName (ie, PCL_Q1), and the variable, a string type, is set to the selected code attribute.

**Figure 3:**
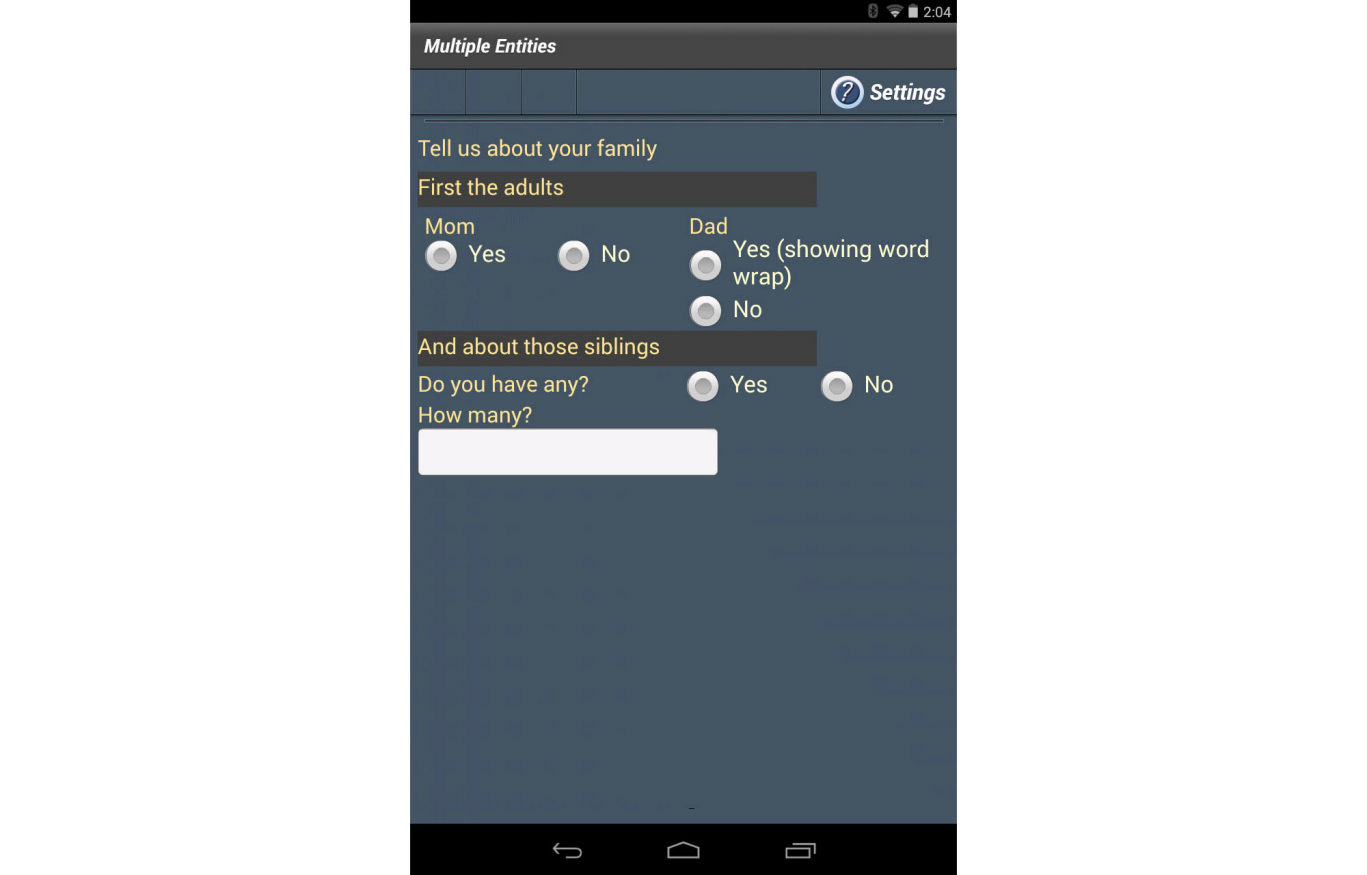
A form containing multiple entities.

Forms are not restricted in the number of entities they can contain. For purposes of this paper, the form example was kept simple. Textbox 2 shows a more complex example showing 4 entities on a form and the use of the vertical and horizontal elements to control user interface layout (Figure 3).

**XML definition.**

<form name="F1">

   <entity name="Q1" type="radio" required="true">

      <text>

         To what extent are you bothered by repeated, disturbing

         memories, thoughts, or images of a stressful experience

         from the past?

      </text>

      <item code="1">Not at all</item>

      <item code="2">A little bit</item>

      <item code="3">Moderately</item>

      <item code="4">Quite a bit</item>

      <item code="5">Extremely</item>

   </entity>

   <logic event="onFormExit">

      <![CDATA[

         if ("{PCL_Q1}">="3") then

            set {PCL_Bcount} = "{PCL_Bcount}" + "1";

      ]]>

   </logic>

</form>

**A more complex example showing 4 entities on a form and the use of the vertical and horizontal elements to control user interface layout.**

<form name="f2" title="Multiple Entities”>

   <vertical styleName="layoutVGroup1">

      <text name="sometextname" save="true" styleName="layoutTextLabel">

         Tell us about your family</text>

      <text styleName="layoutTextLabel2">First the adults</text>

      <horizontal styleName="layoutHGroup100Percent">

         <entity name="mom" layout="itemHorizontal" type="radio"

               styleName="radioWidget" required="true">

            <text>Mom</text>

            <item code="yes">Yes</item>

            <item code="no">No</item>

         </entity>

         <entity name="dad" type="radio">

            <text>Dad</text>

            <item code="yes">Yes (showing word wrap)</item>

            <item code="no">No</item>

         </entity>

      </horizontal>

      <text styleName="layoutTextLabel2">And about those siblings</text>

      <entity name="sib" layout="fullHorizontal" type="radio">

         <text>Do you have any?</text>

         <item code="Y">Yes</item>

         <item code="N">No</item>

      </entity>

      <entity name="numSibs" layout="itemHorizontal" type="text">

         <text>How many?</text>

      </entity>

   </vertical>

</form>

**Figure 4:**
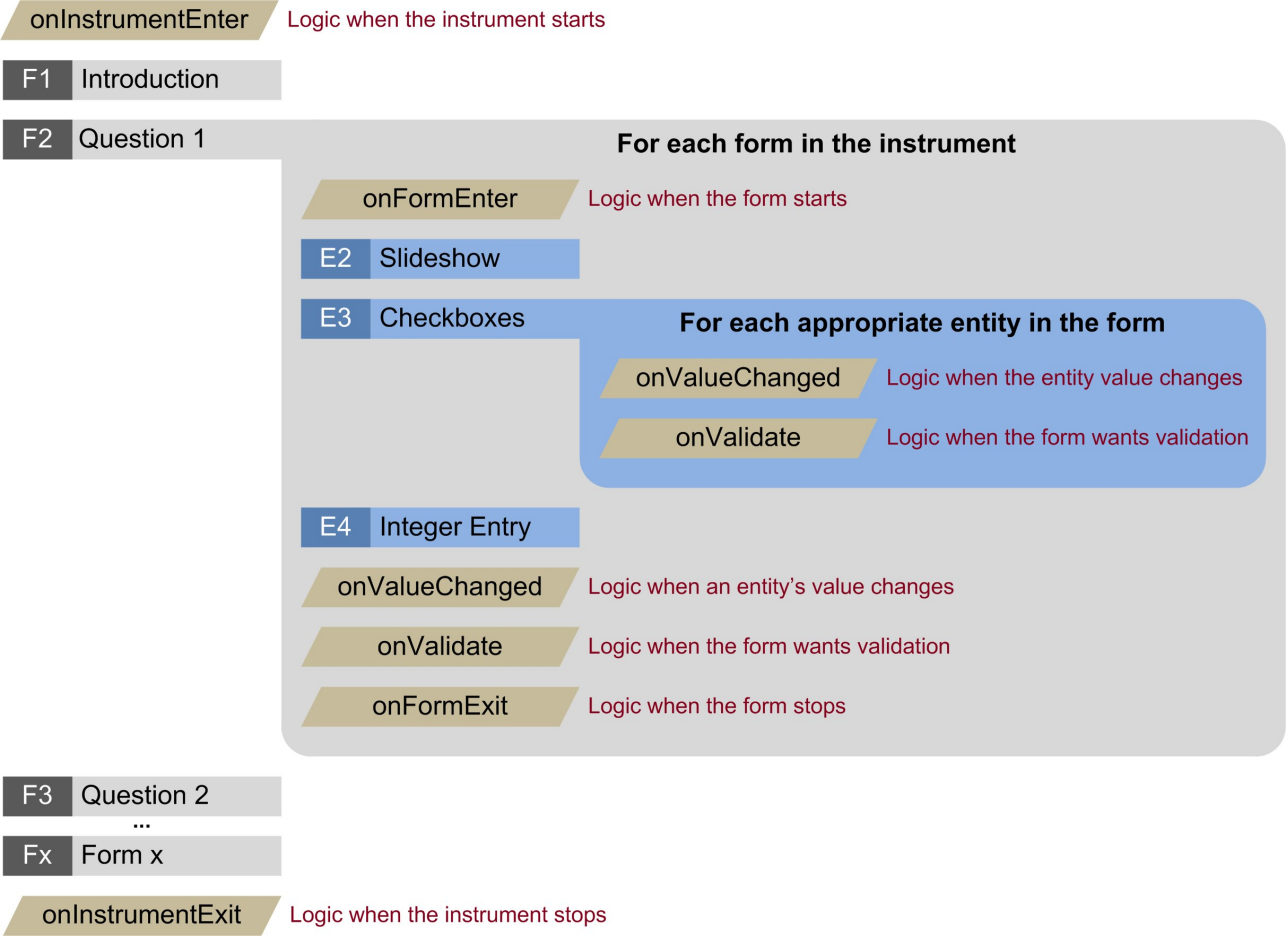
The various events that are triggered during the course of an instrument’s lifetime.

In addition to the instrument and form definitions, business logic is included. There are a number of different events that are triggered during the course of an instrument (Figure 4). Consider again the first form of the Post-Traumatic Stress Disorder Checklist (PCL) instrument. When the user presses the next arrow, the form’s onFormExit code is executed before the next form is displayed. In this case, when the user responds with “Moderately” or higher, the variable PCL_Bcount is incremented by 1.

In Figure 4, the second form (F2) is expanded to highlight the different events. Of particular note are the onValueChanged and onValidate events. The event is first sent to the entity where the data change occurred to provide specific entity-level processing, and then passed up to the form itself where the form can look at all the entities collectively. All scripted logic is written with simple commands (Textbox 3).

Logic within the instrument XML file allows the researcher to decide exactly what validation and skip logic to have, to set initial conditions for instrument variables, and to execute code when the instrument terminates, such as calculating an overall score or saving data to the local secure database. With the instrument completely defined in the XML file, it becomes a reusable object to be shared across apps with the same expected behavior. Other than content used by the instrument, no other dependencies are required.

**Commands for scripted logic.**

set {userHx_ageMonths} = "-1"; // initialize user age

   

if ("{userHx_userID}" == "" && "{userHx_userID_YN}" == "not asked") then

   call message("Are you sure you don't wish to personalize the

            application instrument? If so, hit Next again.");

   

if ("{userHx_ageMonths}" < "0") then

   begin;

      call message("User's birthday must be before today.");

      goto F1_Birthday;

   end;

#### Sensors

**Figure 5:**
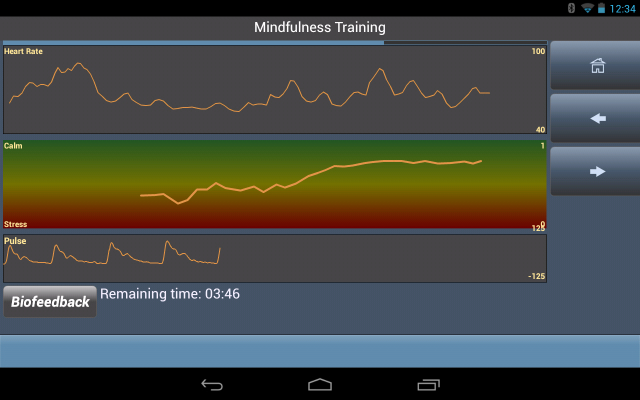
During mindfulness training, an external heart rate monitor captures heart rate data to objectively determine if the user is relaxing or not.

In addition to form-based data entry, the PHIT platform can also collect *objective* data from internal device sensors (eg, global positioning system coordinates) and external Bluetooth sensors (eg, heart rate monitor or fitness accelerometer). In the PHIT for Duty study, where individuals with post-traumatic stress are taught mindfulness exercises for stress reduction, the mobile app uses a heart rate monitor during the mindfulness meditation to calculate heart rate variability (HRV) and graphically show whether the user is achieving a more calm state. This is illustrated in the middle-line chart of Figure 5. Notice the rise in the middle graph after the onset of meditation as the user goes from a stressful state to a calm state.

Whenever sensor data are acquired (eg, the heart pulse rate) and processed to produce a derived measurement (eg, the HRV index), the PHIT software allows for saving interim data at each stage of data processing. Such storage facilitates verification of data-processing algorithms and supports both reanalysis and alternative analysis of raw data at a later date without repeating the data-collection activities. This facilitates exploratory analyses of mHealth data for determining optimal processing methodologies without the expense and effort of repeated field studies, saving a considerable amount of both cost and time.

#### Background Tasks

In addition to user-facing instruments, PHIT provides a means for performing background tasks using instruments without a user interface. Some examples are (1) querying the battery state, (2) uploading data, and (3) retrieving the current global positioning system location. These script instruments, because they run in the background, do not have forms or entities but they do have the onInstrumentEnter and onInstrumentExit events for which custom logic can be written. Such tasks can be developed to execute singularly on demand, or execute repeatedly at a specified interval, like every 5 minutes.

#### Home Screen

Tying it all together, the PHIT platform interprets the instrument definitions and creates the user experience, displaying the appropriate instruments in a task list on the “Home” screen. Attributes of the instrument such as title, description, icon filename, and menu index determine exactly what the user sees and in what order. To reflect a change in state, an XML logic can be written to modify these attributes to highlight changing conditions, alert the user to perform a critical task, or simply change from day to day according to a protocol.

To avoid overwhelming the user with many instruments, instruments can be scheduled. This minimizes burden on the user by cleaning up the user interface and only displaying what is currently relevant. Using the built-in scheduler and the hide and show commands callable from the XML logic, only those tasks appropriate at a certain point in time will be displayed. The PHIT scheduler extends RFC5545, the Internet Calendaring and Scheduling (iCalendar) specification [31], specifically the DTSTART, DTEND, DURATION, and RRULE properties of the VEVENT calendar component. For example, the PHIT scheduler can set an instrument to be scheduled to be on the task list each Friday at 8 am.

#### Virtual Advisor: Tailoring the App for the Users Based on Their Input

PHIT’s iVA is an expert system logic layer where data are *analyzed* and *plans* are created in real time on the mobile device. The iVA tailors its analysis so that the help the user receives is personal and timely, with reanalysis occurring as frequently as the researcher wants it to happen (ie, daily, weekly, or monthly) using the PHIT scheduling function. The iVA program modules are used to stratify health assessments (eg, normal, moderate dysfunction) and to prescribe and schedule self-help activities (eg, exercise, meditation, alcohol reduction) according to the evidence-based criteria provided by the mHealth app researcher.

Consider this example in which a person with a sleep disorder is being evaluated using the Pittsburgh Sleep Quality Index (PSQI) instrument. Upon completing the PSQI, the sleep improvement protocol may recommend the following activities whenever the score exceeds a value of 16:

- Display a slide show on improving the sleeping environment;
- Provide a narrated meditation exercise at bedtime for stress relaxation; and
- Schedule the PSQI instrument to run every 3rd day at 8 am for reassessment until a downward trend is established in the PSQI score.

Just like the instrument logic, iVA logic is defined in XML that is organized by domain for easy reuse. Continuing the aforementioned example, the sleep *assessment* portion of the iVA script is shown in Textbox 4.

**Sleep assessment iVA script.**

<assess domain="sleep">

   <logic>

   <![CDATA[

      // Assess the sleep risk

      if ("{PSQI_complete}" == "true") then begin;

         set {iVA_sleepIntervene} = "false";

         if ("{PSQI_score}"<="7") then set {iVA_sleepRisk} = "1";

         if ("{PSQI_score}">="8" && "{PSQI_score}"<="15") then

            set {iVA_sleepRisk} = "2";

         if ("{PSQI_score}">="16" && "{PSQI_score}"<="19") then

            set {iVA_sleepRisk} = "3";

         if ("{PSQI_score}">="20") then set {iVA_sleepRisk} = "4";

         if ("{iVA_sleepRisk}">"1") then set {iVA_sleepIntervene} = "true";

         set {iVA_scheduleSleep} = "true";

      end;

      if ("{iVA_sleepRisk }" >= "3") then begin;

         // Plan the sleep self-help intervention

         // See the Planning section below for details

   

      end;

   ]]>

   </logic>

</assess>

*Planning* is also handled within the iVA logic. Continuing the previous sleep example where the PSQI score is above 16, the iVA sets the iVA_sleepRisk variable based on the PSQI_score and tests that condition as defined by the protocol in Textbox 5.

**Protocol for testing the PSQI score.**

<assess domain="sleep">

   <logic>

   <![CDATA[

      // Assess the sleep risk

      if ("{PSQI_complete}" == "true") then begin;

   

         // See the Virtual Advisor section above for details

   

      end;

   

      if ("{iVA_sleepRisk }" >= "3") then begin;

   

         // Plan the sleep self-help intervention

   

         // List the sleep environment and mindfulness meditation tasks

         call scheduleTask("sleep%environment");

         call scheduleTask("mindfulness%bodyScan");

   

         // Reschedule the PSQI assessment according to protocol

         call scheduleTask("PSQI", "P1DTA8H", null, "FREQ=DAILY,INTERVAL=3");

      end;

   ]]>

   </logic>

</assess>

The PHIT interventions and activities are implemented using the same scripting and script-processing methods as data-collection instruments; the XML constructs are identical. An intervention might display a series of slides, collect heart rate data during a relaxation exercise, or support behavior modification. For example, a series of images can be combined with audio narration to create an alcohol education module that can be reused across different PHIT apps. Interventions are study and protocol specific and with PHIT’s library of reusable interventions (Table 2), many can be easily tweaked to be content specific for specific intervention needs.

**Table 2:** A partial list of Personal Health Intervention Toolkit (PHIT) interventions used in the PHIT for Duty [8] mobile app.

Category	Self-report data instrument
Stress relaxation	Relaxation breathing
Stress relaxation	Body scan meditation
Stress relaxation	Sitting meditation
Stress relaxation	Walking meditation
Stress relaxation	Loving kindness meditation
Stress relaxation	Heart rate variability biofeedback
Sleep quality	Improving your sleep
Sleep quality	Preparing for sleep
Sleep quality	Personal and environmental factors
Sleep quality	Reclaiming your bedroom
Sleep quality	Sleep smarter skills
Sleep quality	Nightmares
Risk alerts	Post-traumatic stress (Post-Traumatic Stress Disorder Checklist-Military version score > 50)
Risk alerts	Sleep quality (Pittsburgh Sleep Quality Index score > 22)
Risk alerts	Alcohol (Alcohol Use Disorder Identification Test score > 20)
Risk alerts	Anxiety (General Anxiety Disorder-7 score> 15)
Risk alerts	Depression (Patient Health Questionnaire-8 > 10)
Stress management	Arousal control
Stress management	Attention absorption
Alcohol use	Alcohol and stress
Alcohol use	Calories, costs, and consequences
Alcohol use	Drink smarter skills
Alcohol use	Blood alcohol level simulator
Alcohol use	Drinking reduction goals and action plan

#### Logic Processing

The logic written for either instruments or advisor coding is processed by the same logic processor. It supports common programming constructs in a simplified language format. Each logic statement reads like a sentence that ends with a semicolon.

Variables are used to keep track of information in the system, can be accessed globally across XML scripts, and persist until the app terminates. The PHIT naming convention for a variable is <objectName>_<variableName>, which provides a somewhat object-oriented variable naming construct. The PHIT variables are wrapped in “{}” (curly braces) to simplify runtime parsing of the logic code as in Textbox 6.

**PHIT variables in curly braces to simplify runtime parsing.**

if ("{ptHx_status}"=="new") then

   begin;

      set {ptHx_status} = "started";

      set {ptHx_done} = "false";

   end;

When processing this example code statement, PHIT looks up the value of ptHx_status, compares it with the string *new* and if they are equal, sets ptHx_status to the string *started*. The variable ptHx_status refers to the instrument named ptHx (patient history), and the status entity within the ptHx instrument. As no other instrument, or object, may have the same name, the identity of the global variable is assured.

When a variable is evaluated, an attempt is made to determine whether it is a number, and if so, evaluates it as a number. Otherwise, it is treated as a string. If ptHx_age is set to 1, then *if (“{ptHx_age}” <= “2”)* becomes *if (1 <= 2)*. The PHIT logic processor is not rigid; it will automatically convert a quoted number to a real number object when the evaluation is performed, allowing the nonprogrammer who is writing this logic to have more flexibility. Boolean variables remain represented as strings, with “true/false” being the default, designated Boolean values.

A variety of programming statements are supported (Table 3), which are sufficient to meet most programming requirements. These statements are supplemented by the PHIT application programming interface (API), a set of global functions to provide scripted access to frequently used processes such as data conversion, database storage, and retrieval, playing sounds for effects and notifications, displaying pop-up messages, and formatting number variables as strings.

**Table 3:** A sample of Personal Health Intervention Toolkit logic statements and application programming interface function calls.

Statement	Example
Set a variable	set {ptHx_age} = “15”; set {ptHX_gender} = “M”;
Call a function	set {age_m} = call calculateAgeInMonths("{ptHx_birthday}"); call Message("Patient birthday must be before today."); set {iVA_height}=call formatNumber({iVA_height}, 0);
Conditional logic	if ("{ptHx_ageMonths}">="0" && "{ptHx_ageMonths}"<="48" && "{iVA_isExposed}")then set {iVA_tobaccoCode}="Under4Exposure";
Skip logic	if ("{ptHx_ptID}"=="") then goto F0_PtID;
Exit the instrument	exit;
Move to next form in form stack	if (("{ptHx_today}"-"{ptHx_birthday}")<"{ptHx_one_week}") then nextForm;
Nested ifs and while	set {randytest_collectId1} = call generateCollectionId(); if ("{randytest_collectId1}">="0") then begin; set {iVA_alcoholInstr} = "CAGE"; set {iVA_alcoholInstrWording} = "past two weeks"; if ("{randytest_collectId1}" == "0") then begin; set {iVA_another} = "foobar"; end; set {iVA_alcoholOnQueue} = "foobar"; set {iVA_alcoholStatus} = "monitoringScheduled"; set {iVA_i} = 0; while ({iVA_i} < 3) begin; set {iVA_i} = {iVA_i} + 1; if ("{iVA_i}" == "1") then set {randytest_zzzz} = "zzzz"; end; end; set {randytest_collectId2} = call generateCollectionId(); var {randytest_abar} = "";
Statement blocks	begin; set {ptHx_status} = "started"; set {ptHx_done} = "false"; end;

To analyze historical trends, the API provides query functions to the local database as the following shows:

- “findLatest” retrieves the last saved value
- “findLatestN” retrieves the last N saved values
- “findByDate” retrieves values based on data range (eg, everything between June and September)
- “findByPlacement” retrieves values based on placement range (eg, everything between the fourth and eighth save)

Should a particularly complex XML script cause performance problems due to runtime compilation, you may recode the XML script in native code as an API function as “isPSQITrendingDownward.”

Although not common, any native code you write is automatically compiled into your PHIT app when the app is built.

### Creating Your Own PHIT App: How the Pieces Fit Together

**Figure 6:**
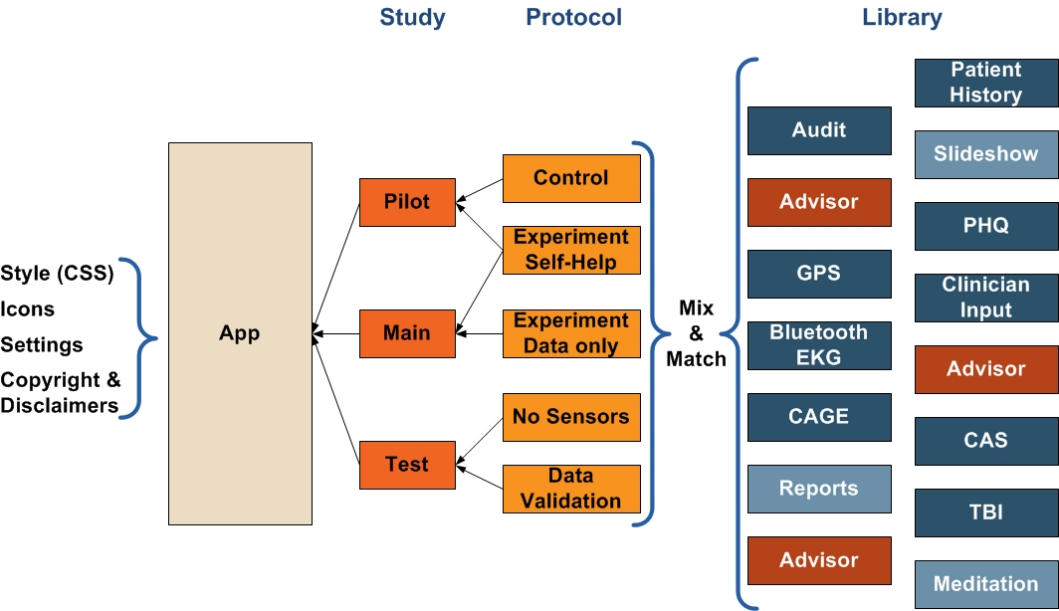
Personal Health Intervention Toolkit app configuration.

Unlike most mHealth apps, PHIT apps can be configured to support specific research requirements including multiple-treatment research studies. Using XML configuration files, you further define the app into studies and protocols. Each PHIT study contains one or more protocols and each protocol contains the instruments, virtual advisor, and interventions to be used for that protocol (Figure 6). In this way, different treatment groups are automatically embodied in the app.

With customization at the protocol level, each protocol in a study can have very different instruments, interventions, and virtual advisor; thus, creating a very different app. One example is a study in which Protocol A collects data and runs the virtual advisor to display appropriate interventions based on assessment scores, whereas Protocol B may merely collect data and have neither a virtual advisor nor any interventions.

### Data Storage

Instrument-defined entity data are optionally saved in a local database with each record tagged with project id, study id, protocol id, case id, observation id, and a date-timestamp. Although not required, the PHIT platform supports, and strongly recommends that all data be stored using encryption to ensure data privacy. If the database is encrypted, PHIT automatically enforces the use of a password to access the app.

Although apps usually require a single database, the PHIT framework allows for multiple databases, including both create, update, and delete and read-only databases. A read-only database might be a lookup table to support app requirements, such as percentile growth chart for a pediatric wellness check [32].

### Data Upload

In addition to the local data store, PHIT provides a data upload capability to a backend database. The following two upload options are available: (1) a user-initiated upload function, which initiates transfer and provides status feedback via an upload progress bar, and (2) a utility instrument for uploading to the server in the background. Because instruments can be scheduled, the mobile app can be configured to initiate a background data transfer on a prescheduled time, such as once a day at 12 am, thereby providing little disruption to the user. Of course, the app must be running on the device for this to occur. For privacy, data are uploaded over hypertext transfer protocol secure (HTTPS) with the option to encrypt the data before being uploaded over HTTPS, providing a doubly encrypted upload. Uploading data to a central server is an optional feature, which is most useful for research studies; however, use of a central data store is not a required element of a PHIT app.

To accommodate the typical software development process, three-dimensional different upload URLs can be specified to mimic the different phases of software development:

- Development;
- Test/staging; and
- Production.

This has the benefit of not polluting the production database with test data.

When merged into one dataset on a backend data server, data can be visualized, studied, and extracted. Access to the project data is controlled via an access control list to ensure data privacy. By default, PHIT uploads no personally identifiable information (PII) ensuring that all data are deidentified but allowing for data to be reported up to the case id level. However, it is up to the app development team creating the mobile app to ensure that PII data are not misidentified, leading to accidental upload.

### User Interface Customization

**Figure 7:**
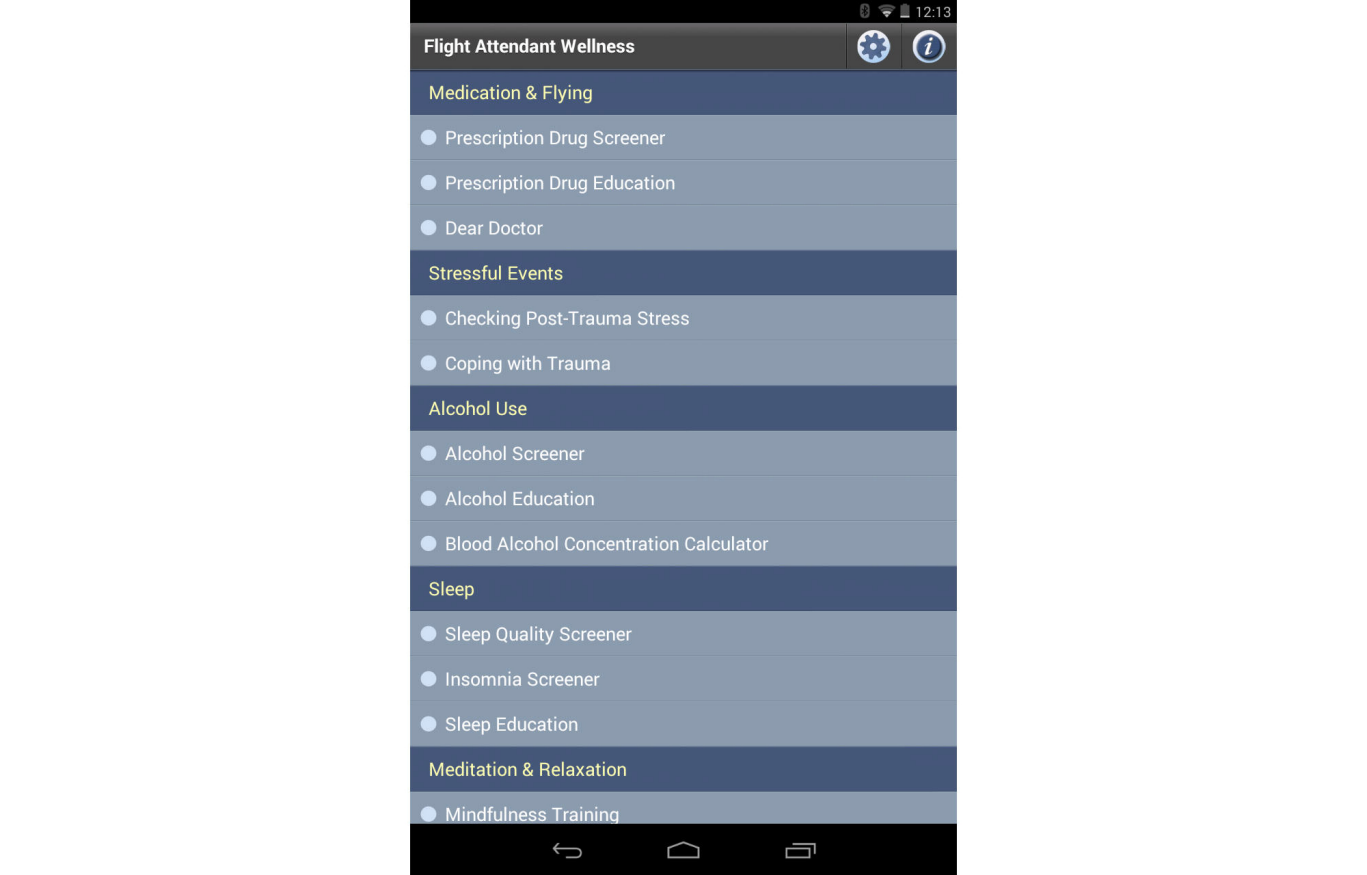
Flight Attendant Wellness app home screen.

**Figure 8:**
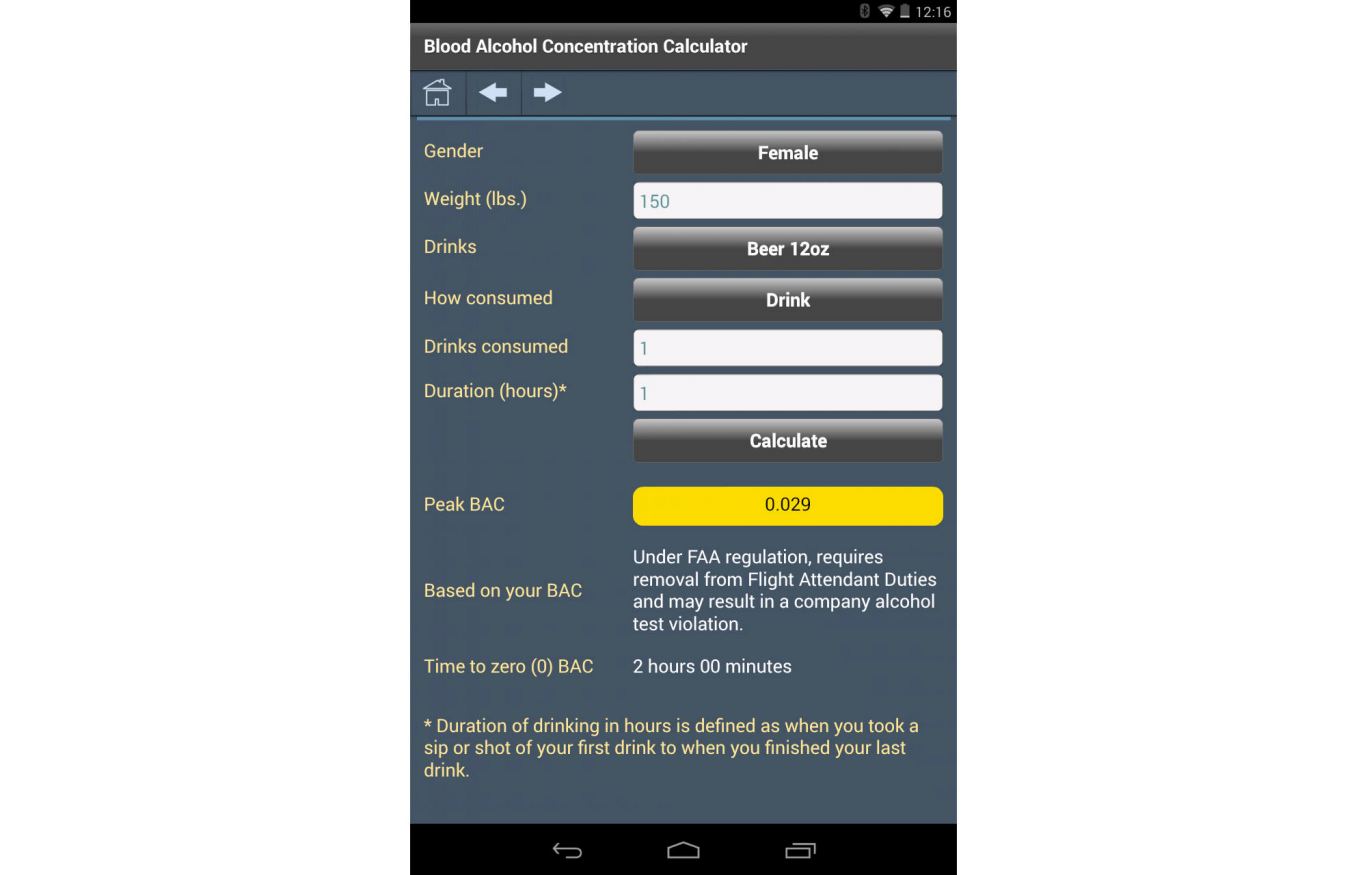
Flight Attendant Wellness app blood alcohol concentration calculator.

**Figure 9:**
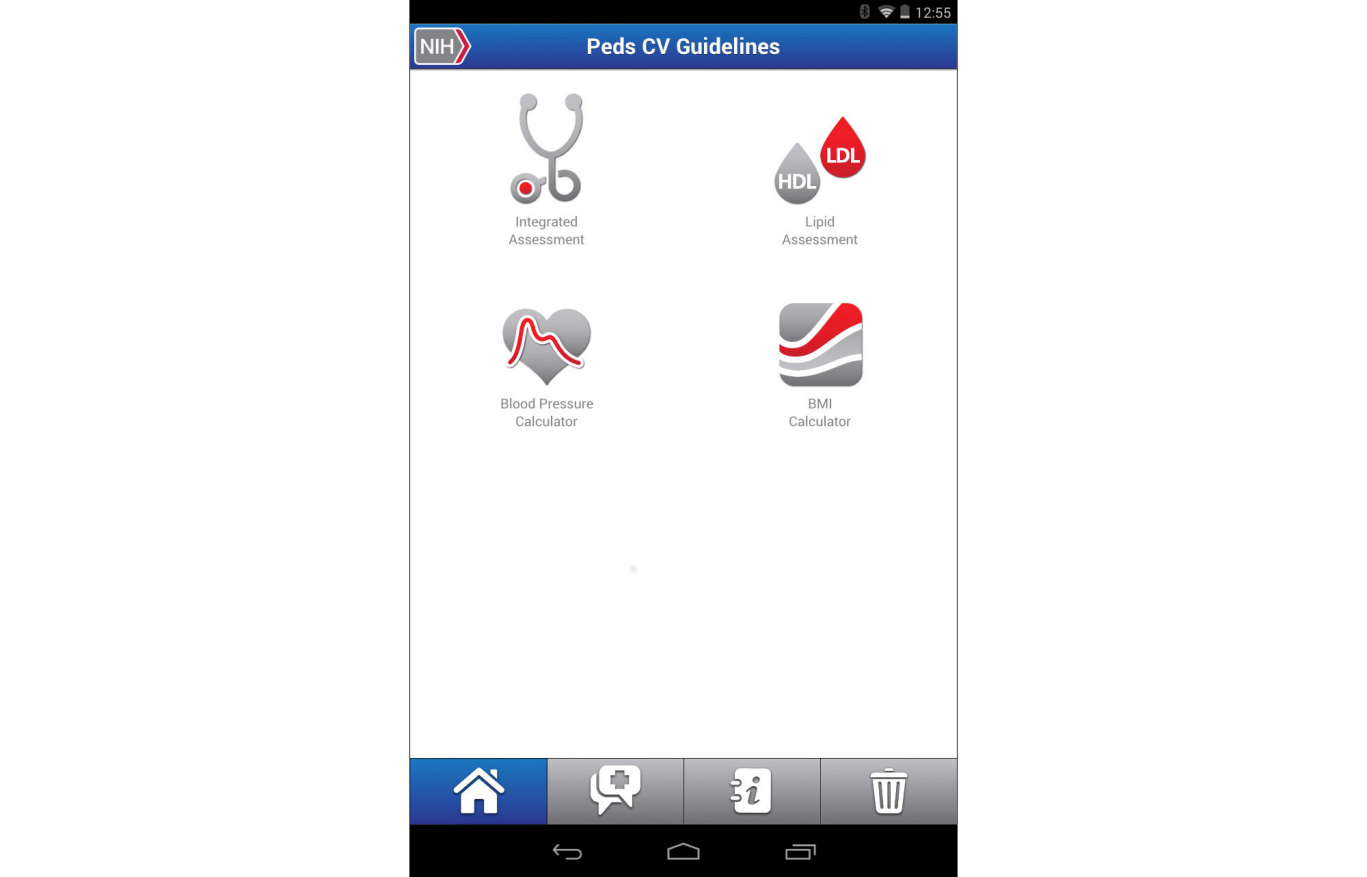
Clinical decision support tool for Pediatric Cardiovascular Risk Reduction app home screen.

**Figure 10:**
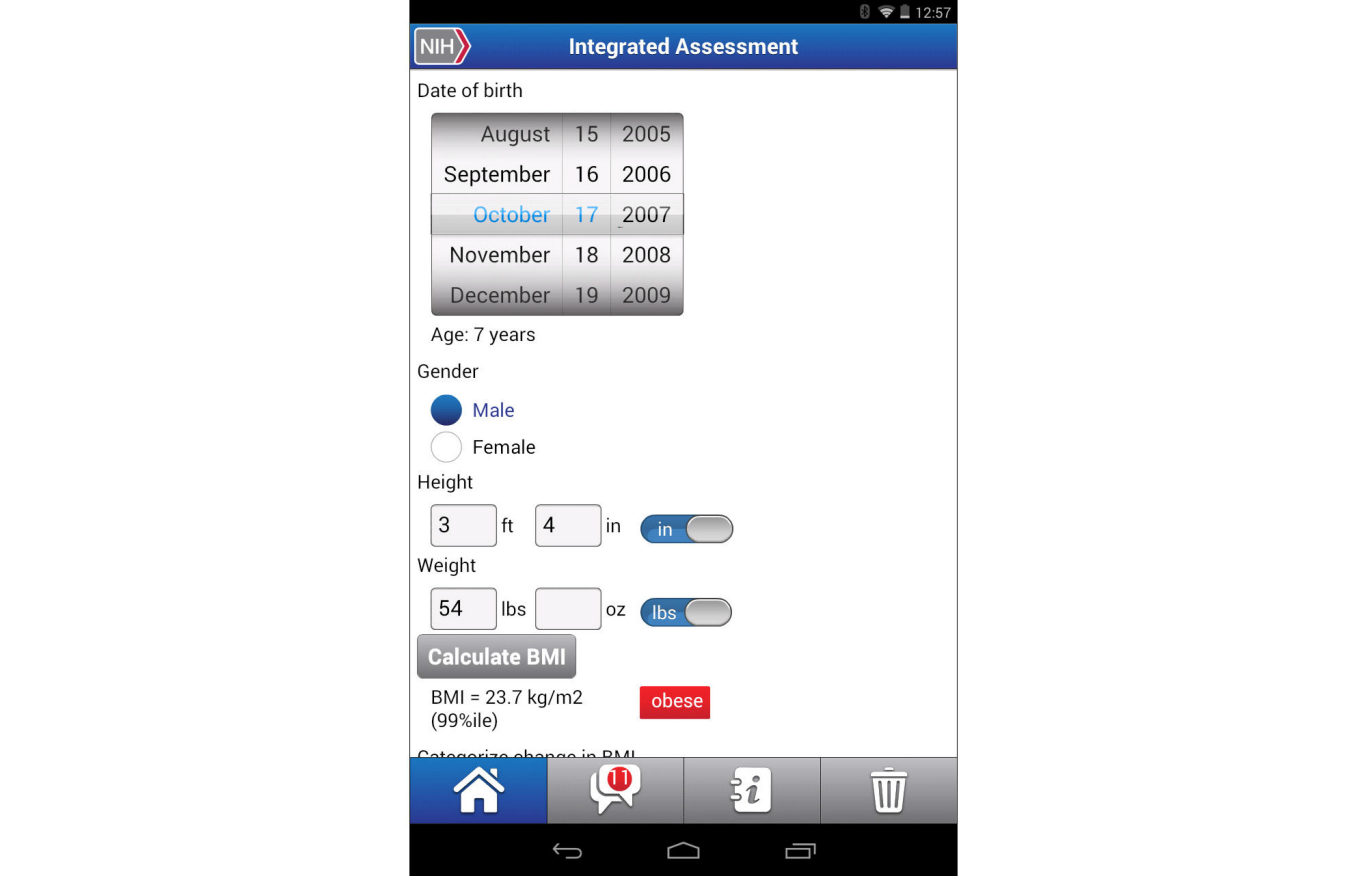
Clinical decision support tool for Pediatric Cardiovascular Risk Reduction app integrated assessment screen.

Mobile app developers need to address differences in screen size and density, and differences across mobile device software platforms. Each platform has a distinct user experience and human interface guidelines. The technology PHIT is built on uses a neutral look and feel across these platforms. The appearance of the mobile app can be changed through the use of cascading style sheets (CSS), custom skins, and icons, giving each app its own unique look and feel (Figures 7-10).

### What Skills Do You Need?

With minimal customization of the app, only knowledge of XML and workflow logic is required to implement most mHealth apps. Custom apps require various software engineering skills. Some of these optional skills are as follows:

- Mobile app development familiarity;
- Graphics, video, and audio content design;
- CSS;
- Adobe Integrated Runtime, Apache Flex, and ActionScript;
- Java for Android native code;
- Objective-C for iOS native code;
- Encryption; and
- HTTP programming

## Results

### Overview

To date, we have completed 4 mobile apps and 1 desktop app using the PHIT platform. Several additional apps are in development and a half-dozen are in the concept phase. We have found that the time from concept to completion and the cost of implementation were substantially reduced in the later projects, compared with the initial work attesting to the flexibility of the PHIT platform and the reusability of developed components. Examples of our completed apps are as follows:

### PHIT for Duty, a Personal Health Intervention Tool for Psychological Health and Traumatic Brain Injury

PHIT for Duty, deployed on Android devices, includes over 30 psychometric, personal/medical history, trauma exposure, and other data-collection instruments and evaluations [8]. Self-help interventions have been developed for stress, sleep problems, and alcohol abuse, including multimedia health information modules, stress relaxation exercises, and cognitive behavior therapies for sleep and alcohol.

### Clinical Decision Support for Cardiovascular Health and Risk Reduction in Children and Adolescents

This app implements data collection, risk assessment, and intervention recommendation requirements of a subset of the Guidelines on Pediatric Cardiovascular Health and Risk Reduction [33]. The mobile app, intended for use by pediatricians aids to facilitate their use of the guidelines in daily clinical practice, is easy to use, and available for both Android and iOS devices.

### Pre-Deployment Stress Inoculation Training (PRESIT)

This is a desktop app for training in stress reduction techniques as a preventative measure for reducing incidence of post-traumatic stress in service men and women.

### ActiSleep

An app used for collecting research data in a study of sleep habits, sleep quality, and substance use in teenagers. The app includes daily diaries for prebedtime activities, substance use, and sleep quality. It also provides step-by-step multimedia instructions to enable participants to carry out biosample collection (ie, saliva) and facilitate use of a sleep activity monitor, thereby maintaining data quality in these ancillary data-collection processes.

### Flight Attendant Wellness

An app providing screeners and education to support the prevention of prescription drug abuse, the federal model drug-free workplace, and the Workplace Prevention Research initiative.

Use of the PHIT framework for your apps does not limit you in how you distribute your apps nor do they require any oversight or verification. You are in complete control in distributing your PHIT app, whether it is made available via a public app store or a private distribution. The PHIT for Duty and ActiSleep apps are both for private research studies with a private distribution. The Flight Attendant Wellness app and the Clinical Decision Support for Cardiovascular Health and Risk Reduction in Children and Adolescents app are in the process of being made publically available and should be in the app stores soon.

## Discussion

The PHIT framework has proven to be an extensible, reusable, and reconfigurable technology that facilitates mobile data collection and health intervention research. In addition to specific project requirements to enhance the platform, plans are to grow the library of instruments and interventions, add simple texting service prompting and notification, provide distributed advisor processing on the backend, and improve the Bluetooth layer for access to sensors, including wearable sensors.

## Abbreviations

API: application programming interface
CSS: cascading style sheets
HRV: heart rate variability
HTTPS: hypertext transfer protocol secure
iVA: intelligent virtual advisor
PCL: Post-Traumatic Stress Disorder Checklist
PHIT: Personal Health Intervention Toolkit
PII: personally identifiable information
PRESIT: Pre-Deployment Stress Inoculation Training
PSQI: Pittsburgh Sleep Quality Index
PTSD: post-traumatic stress disorder
XML: extensible markup language
